# Supplementary material for: Pregnant women’s and health workers’ perceptions and experiences on the Rwandan ANC digital module intervention at selected health centres
Source: PLOS Digit Health. 2026 Feb 24;5(2):e0001264. doi: 10.1371/journal.pdig.0001264 (PMC12931765; doi:10.1371/journal.pdig.0001264)
Supplement: S1 Text — (DOCX) [file pdig.0001264.s001.docx]

**Focus Group discussion guide for pregnant women regarding the adapted Rwanda ANC Digital Module**

**Introduction**

Welcome and thank you for being here today. My name is “_____________” and I am part of the study team conducting the implementation science research on the new adapted ANC module based on the 2016 WHO revised ANC guidelines in the provision of antenatal care. This study aims to better understand how the adapted Rwanda antenatal care (ANC) digital module can support health workers and lead to better health services and outcomes for women and children. The purpose of this focus group discussion is to get your feedback on ANC digital module. We want to understand what works and what doesn’t work. Your opinions and perceptions are important to us. I will guide the conversation by asking questions. There are no right or wrong answers to these questions. If you wish, you can also respond to each other’s comments, like you would in an ordinary conversation. This focus group is anonymous and confidential. We would like to audio record this conversation so that we do not miss important points discussed but instead focus on the discussion. The recording will only be used to make sure our notes are correct and will not be heard by anyone outside of this research.

**Profile information for each participant of FGD**

| No | Age | Marital status  (Single; Married; Divorced; Separated; co- habiting; Widow) | Highest grade (None, Primary, Secondary and Tertiary) | Number of pregnancies | Number of ANC visits at the Facility |
| --- | --- | --- | --- | --- | --- |
| 1. |  |  |  |  |  |
| 2. |  |  |  |  |  |
| 3. |  |  |  |  |  |
| 4. |  |  |  |  |  |
| 5. |  |  |  |  |  |
| 6. |  |  |  |  |  |
| 7. |  |  |  |  |  |
| 8. |  |  |  |  |  |
| 9 |  |  |  |  |  |
| 10 |  |  |  |  |  |

**Discussions**

1. Did you observe a difference in service provision when healthcare workers used the cellphone?

**Probe:**

- Did the provider explained the use of the tablet?
- Were you able to ask him/her question about it?
- Do you understand what the cellphone is being used for?
- How did the use of the cellphone impact the communication or interaction with the healthcare worker?

1. What aspects of the health worker's use of the cellphone during your ANC contact did you appreciate or like? Probe: Did the cellphone make the consultation feel more organized or efficient? Did the use of the cellphone improve the clarity of the information you received?

Did the cellphone usage allow the provider to address your questions or concerns more effectively? How did the cellphone contribute to your overall confidence in the care you received?

1. What aspects of the health worker's use of the cellphone during your ANC contact did you not appreciate or dislike? **Probe:**
   - Whether the cellphone usage make the provider seem distracted or less attentive.
   - Was the consultation longer or shorter than expected because of the cellphone use?
   - Did you feel that the use of the cellphone affected the privacy or confidentiality of your care?
   - Were there moments when the provider seemed to struggle with the technology, and did that affect your experience?
2. If this is not your first visit pregnancy, did the health worker’s use of the cellphone impact your antenatal care visit? If yes how?

**Probe:** The length of your contact? The way the provider treated you? The provider’s attention?

**Conclusion**

Would you like to discuss anything else related to your antenatal care experience that we have not discussed?

Thank you very much for your time today. Everything we have discussed here remains confidential and will not be spoken outside of this group discussion.
