# Supplementary material for: Pregnant women’s and health workers’ perceptions and experiences on the Rwandan ANC digital module intervention at selected health centres
Source: PLOS Digit Health. 2026 Feb 24;5(2):e0001264. doi: 10.1371/journal.pdig.0001264 (PMC12931765; doi:10.1371/journal.pdig.0001264)
Supplement: S2 Text — (DOCX) [file pdig.0001264.s002.docx]

**Key informant interview guide for heads of health centers regarding the Rwandan ANC Digital Module**

**Introduction**

Welcome and thank you for being here today. My name is _________, and I am part of the study team conducting implementation science research on the Rwanda adapted ANC guidelines based on the 2016 WHO revised ANC guidelines for a positive pregnancy experience. The purpose of this interview is to gather your feedback on the implementation of the adapted digital ANC module. We would like to understand what works for you and your team at this facility, as well as what does not work well, because you have a better understanding of what works regarding the ANC digital tool. I will guide the conversation by asking questions that you can respond to. There are no right or wrong answers to these questions. I would like to audio record this conversation so that we don’t have to focus on notetaking during the discussion.

Let me pause for a moment to address any questions you may have. Do you have any questions?

**Key characteristics of the participant**

| District Name |  |
| --- | --- |
| Interviewer ID |  |
| Date of KII |  |
| KII Number |  |
| Start time of KII |  |
| End time of KII |  |
| Highest Educational Level attained |  |
| Age in years |  |
| Length of time working at this position: |  |

**Discussions/Questions**

I would now like to ask you about your experience with health providers using the digital module at this facility. As you may be aware as part of this implementation research, health providers were trained to use a digitalized module customized to the Rwandan context.

  1. Please describe you and your staff’s overall experience using the Rwanda ANC Digital Module. **Probe further**

- *What did you find useful about your staff using the Rwanda ANC digital module?*
- *What did you find difficult about your staff using the Rwanda ANC digital module?*
- *What parts of the Rwanda ANC digital Module were most helpful for you and your staff?*
- *How does the Rwanda ANC Digital Module affect data collection and quality at your facility?*
- *What supervision needs do you think the Rwanda ANC Digital Module will entail?*
- *What are some of the most commonly reported challenges by HF during the use of the NAMAI digital module?*
- *Overall, do you think that the NAMAI digital module should be adopted by the country/integrated in the digital ecosystem?*

1. Please describe you and your staff’s overall training experience on how to use the Rwanda ANC digital Module. **Probe further**

- *Follow up: What did you find useful about the training?*
- *Follow up: Was there anything you missed during the training?*
- *What did you think of the supervisory tools for the Rwandan ANC digital module?*

1. Do you think in the long term it will be possible to use the Rwandan ANC digital module for providing antenatal care? **Probe:**

- Quality of training received, resource availability, support from supervisor(s) / facility staff / peers, treatment and referral services, location of ANC session, facilities available
- *What parts of the Rwanda ANC Digital Module were most helpful for you?*

1. How has the digital module improved impacted how patients are attended to and followed up?

***Probe:*** *wait times? Level of attention from you the provider? Etc.*

1. What additions or changes would you suggest to improve experience of both providers and clients during the implementation of the ANC digital module?

***Probe:*** *health providers training; need in equipment and/or infrastructure (i.e electricity, internet access), regular supervision, linkage between health post and health center, etc.*

**iii) Conclusion**

Is there anything else you would like to add? Or anything you thought would come up in our discussion but didn’t?

- This has been a very successful discussion. Your opinions are valuable. We hope you have found the discussion interesting.
- I would like to remind you that any comments and feedback are confidential and anything you share will help improve ANC services for women in Rwanda.
  - Thank you for your participation and thank you very much for your time.
