# Supplementary material for: Pregnant women’s and health workers’ perceptions and experiences on the Rwandan ANC digital module intervention at selected health centres
Source: PLOS Digit Health. 2026 Feb 24;5(2):e0001264. doi: 10.1371/journal.pdig.0001264 (PMC12931765; doi:10.1371/journal.pdig.0001264)
Supplement: S3 Text — (DOCX) [file pdig.0001264.s003.docx]

**Key informant interview guide for nurses providing ANC services regarding the Rwandan ANC Digital Module**

**Introduction**

Welcome and thank you for being here today. My name is _________, and I am part of the study team conducting implementation science research on the Rwanda adapted ANC guidelines based on the 2016 WHO revised ANC guidelines for a positive pregnancy experience. The purpose of this interview is to gather your feedback on the implementation of the adapted digital ANC module. We would like to understand what works for you and your team at this facility, as well as what does not work well, because you have a better understanding of what works regarding the ANC digital tool. I will guide the conversation by asking questions that you can respond to. There are no right or wrong answers to these questions. I would like to audio record this conversation so that we don’t have to focus on notetaking during the discussion.

Let me pause for a moment to address any questions you may have. Do you have any questions?

**Key characteristics of the participant**

| District Name |  |
| --- | --- |
| Interviewer ID |  |
| Date of KII |  |
| KII Number |  |
| Start time of KII |  |
| End time of KII |  |
| Highest educational level attained |  |
| Age in years |  |
| Length of time working at this position: |  |

**Discussions/Questions**

1. **Please describe your overall experience with the NAMAI digital system you have been using for antenatal care.**

**Probe:**

- 1. How was the NAMAI digital system introduced to you?
  2. What do you enjoy or like about it?
  3. What do you find easy about using it? What was the most useful part of the NAMAI digital module?
  4. Did you have to re-arrange clinical flows/develop strategies to integrate the module within your facility?
  5. How long did it take you to be familiar/comfortable with the system?
  6. Were there some of the parts/areas that took you longer? If yes, how?
  7. Did you receive any training? Please tell us about the training. What was made clearer with the training? What did you still have questions about? How was the Quality of training received?

1. **How were your interactions with the digital system changed? This can be changes in the data you enter, prompts, and alerts you receive.**

**Probe:**

- 1. How does the updated system compare to how you previously used the digital system or any other systems (paper)? What are the major changes you have observed?
  2. How do you feel about this change? What are some of the challenges you faced?
  3. What changes were most helpful or make your work easier?
  4. What changes were least helpful or have made your work harder?
  5. What are some reasons why or situations in which you might not want to use the NAMAI ANC digital system?
  6. What changes would you recommend to improve it? Are there parts the can be shortened/combined?

1. **Please describe how the NAMAI digital system affected your interactions with new or existing clients.**

**Probe:**

- 1. How did it change anything about the rapport you have with clients (pregnant women)?
  2. How did it change anything about the trust you feel clients (pregnant women) have in you?
  3. Were there any changes made to your clinical processes to accommodate the use of the tool?
  4. Was the SMS feature useful in reminding clients to attend ANC? Approximately how many more clients did you see per due to SMS? Any general feedback you have on the messages?

1. **What are the main challenges you have faced in implementing the ANC Digital Module?**

**Probe:**

1. Have there been any issues with device availability, maintenance, or connectivity?
2. How has the integration of the module into existing workflows or systems been managed?
3. Are there challenges related to training, digital literacy, or resistance to adopting new technology among staff?
4. Has the module increased or decreased your workload? How does this affect care delivery? **Probe:** wait times? Level of attention from you the provider?
5. Were there some of the components/functionalities that were not useful?
6. How do patients respond to the use of digital tools in their care?
7. What recommendations would you make to improve the ANC Digital Module, and what opportunities or lessons can be drawn from your experience?

**Conclusion**

Is there anything else you would like to add? Or anything you thought would come up in our discussion but didn’t?

Thank you so much for your time.
