# Supplementary material for: Pregnant women’s and health workers’ perceptions and experiences on the Rwandan ANC digital module intervention at selected health centres
Source: PLOS Digit Health. 2026 Feb 24;5(2):e0001264. doi: 10.1371/journal.pdig.0001264 (PMC12931765; doi:10.1371/journal.pdig.0001264)
Supplement: Attachment — Submitted filename: PLOS Digital Health Manuscript_ Review.pdf [file pdig.0001264.s004.pdf]

Reviewer Report – PLOS Digital Health

Manuscript: *Pregnant women's and health workers' perceptions and experiences on the Rwandan ANC digital module intervention at selected health centres*
Manuscript ID: PDIG-D-25-01089

1. Overall Assessment

This manuscript presents a well-conducted qualitative study exploring health workers' and pregnant women's experiences with Rwanda's WHO SMART–aligned digital ANC module implemented under the NAMAI study. The paper is clearly written, methodologically sound, and grounded in an important implementation context. The findings are coherent and align with existing evidence on digital maternal health tools in low-resource settings.

The study offers useful, policy-relevant insights for countries implementing digital decision-support tools within routine ANC services. Only **minor revisions** are required to strengthen clarity, positioning within the digital health literature, and analytic transparency.

2. Minor Comments and Suggested Revisions

2.1 Clarifying the Digital Health Contribution

The manuscript would benefit from a slightly clearer articulation of its contribution to the digital health literature, particularly to distinguish this study from prior NAMAI- or SMART-related publications.

Suggested clarification:

- Briefly highlight what this study adds in terms of *user experience, workflow integration, or real-world implementation lessons* beyond previous evaluations.
- Explicitly position the findings as implementation learning relevant for digital tool optimization and scale-up, rather than as novel effectiveness evidence.

2.2 Strengthening the Discussion on System Integration

The discussion touches on DHIS2 integration, supervision, and dual paper–digital use, which are important system-level issues. These points could be strengthened with minimal expansion.

Suggested clarification:

- Add brief reflections on how policy, reporting requirements, or regulatory factors may contribute to continued paper-based documentation.
- Clarify implications for transitioning from demonstration projects to routine national implementation.

3. Minor Editorial and Presentation Issues

1. Minor grammatical and typographical errors remain (e.g., “algorism” instead of “algorithm”)
2. Tables 1 and 2 should use consistent statistical terminology (e.g., “Mean” instead of “Average”).
