# Supplementary material for: Pregnant women’s and health workers’ perceptions and experiences on the Rwandan ANC digital module intervention at selected health centres
Source: PLOS Digit Health. 2026 Feb 24;5(2):e0001264. doi: 10.1371/journal.pdig.0001264 (PMC12931765; doi:10.1371/journal.pdig.0001264)
Supplement: Attachment — Submitted filename: Response to Reviewers.pdf [file pdig.0001264.s006.pdf]

Dear: Editor

First and foremost, we sincerely acknowledge the reviewers' insightful and constructive comments. Their feedback has been invaluable in enhancing the quality of our manuscript. We have carefully addressed the comments in a detailed point-to-point manner (See below).

**Editor feedback**	**Response**
1. Positioning within the digital health literature Reviewers noted that the Background and Discussion would benefit from more clearly situating this study within the broader digital health and maternal health literature, particularly in sub-Saharan Africa. Please:	
• Include a paragraph contextualizing the role of digital health tools in improving ANC uptake and quality in sub-Saharan Africa.	A paragraph on the role of digital health tools in improving ANC uptake and quality in sub-Saharan Africa is added on lines 106 to 113.
• Clarify the specific evidence gap this study addresses. Distinguish what this paper adds beyond prior NAMAI - or SMART - related publications, particularly in terms of user experience, workflow integration, or real-world implementation learning.	The gap was revised according to the comment as follows: *Although digital ANC modules have demonstrated potential to improve service utilization and maternal outcomes in sub-Saharan Africa, evidence remains limited regarding user experiences, workflow integration, and real-world implementation. A qualitative approach is therefore warranted to explore the experiences and perceptions of pregnant women as end users and healthcare providers involved in the use and implementation of digital ANC systems. Thus, this study aimed to explore the experiences and perceptions of health workers and pregnant women regarding the Rwanda digital ANC module, an intervention part of the NAMAI study, in selected health centres.*

2. Clarifying the digital health contribution and interpretation. Several findings align with broader digital health implementation literature. Reviewers recommend:<br>• More explicitly framing the results as digital health design and implementation lessons rather than effectiveness evidence. Tightening language in a few places to clearly distinguish perceived improvements from objectively measured outcomes.<br>• Including a brief synthesis in the Discussion that highlights implications for system speed, offline functionality, supervision models, and workflow integration.	In line with the qualitative nature of the study, the results section was revised to emphasize digital health design and implementation lessons, focusing on perceived improvements rather than objectively measured outcomes.<br><br>This is addressed on lines from 452 to 462.
3. System integration, scale-up, and implementation considerations. Reviewers raised several related points that should be addressed with modest expansion:<br>• Strengthen discussion of DHIS2 integration, supervision, and continued paper–digital dual use. Add brief reflections on how policy, reporting requirements, or regulatory factors may contribute to continued paper-based documentation. Clarify implications for transitioning from demonstration projects to routine national implementation, including workload considerations and stakeholder involvement (e.g., Ministry of Health).<br>• Explain why some participants did not receive text messages and whether this has implications for nationwide rollout.	These are addressed on lines from 452 to 462.<br><br>Following a consultation with the technical team, a configuration issue in the digital system was preventing SMS reminders from being sent to

	pregnant women. However, following a data migration process, all women began receiving SMS reminders.
4. Methods clarification and analytic transparency. To improve clarity and replicability, please:<br>• Clarify whether the thematic analysis followed an inductive or theory-driven approach within the Braun and Clarke framework.<br>• Consider making interview and focus group discussion guides available as supplementary materials, if feasible.	The thematic analysis was conducted following Braun and Clarke's framework using an inductive approach. Themes were generated from the data without the application of a pre-existing theoretical model.<br><br>The interview guide and group discussion guide are attached as supplementary files.
5. Editorial, language, and data reporting issues. Reviewers identified several minor but important presentation issues:<br>• Address grammatical and typographical errors throughout the manuscript (e.g., "algorism" should be "algorithm"). Ensure consistent statistical terminology in Tables 1 and 2 (e.g., "Mean" rather than "Average"). Proof-read all sections to improve clarity and readability.<br>• Confirm that the Data Availability Statement fully complies with PLOS policy and clarify any inconsistencies noted by reviewers.	We have proof-read and addressed the grammatical and typographical errors.<br><br>This was revised as indicated above.
**Reviewer 1**	
**Background:**<br>**Major comment:**	

The focus of the paper is on the ANC digital module intervention. The Background section should situate the role of digital health tools in promoting healthcare/ANC uptake (in sub-Saharan Africa). I recommend including a paragraph on this and clarifying the knowledge gap that the research is looking to fill.<br><br>**Minor comments:**<br>1. Lines 67-75 (the first paragraph) presents a lot of statistics including multiple regions (sub-Saharan Africa, Central and Southern Asia). The authors can make this more succinct and present only the most important figures to make their point.	A paragraph on the role of digital health tools in improving ANC uptake and quality in sub-Saharan Africa is added on lines from 106 to 113.<br><br>Thank you for the comment, we have revised the paragraph.
2. Line 77-78: Reference [8] is used as evidence to support the statement “maternal and neonatal deaths can be reduced significantly further, through provision of quality care services before, during and after childbirth” – yet the reference is to a protocol. I suggest using alternative references with evidence to support this.	Thank you for this observation, now we replaced with alternative relevant supporting evidence.
**Methods:**<br>The authors explain the site selection, participant selection, data collection and data analysis processes. Minor comments:<br><br>1. The Methods section has generally been written in passive voice. I suggest the authors consider using active voice to minimize ambiguity as to who was performing the different tasks.	 <br><br><br>This comment is well noted, and we have applied active voice throughout where appropriate.

2. Line 169: The authors state that they followed the Braun and Clarke thematic analysis process. Can they clarify whether they conducted Inductive analysis or Theoretic analysis of the data.<br><br>3. Will the interview/FGD guides be made available in the supplementary materials? This can help in understanding the responses and replicating the research elsewhere.	The thematic analysis was conducted following Braun and Clarke's framework using an inductive approach.<br><br>The interview guide and group discussion guide are attached as supplementary files.
**Reviewer 2**	
To further strengthen the manuscript, the authors may consider the following refinements:<br>• **Clarify the digital health learning:** Many of the findings resonate with broader digital health implementation literature. A brief synthesis in the discussion that explicitly frames these results as design and implementation lessons (e.g., implications for system speed, offline functionality, supervision models, and workflow integration) would enhance the manuscript's relevance for digital health audiences.<br>• **Interpretive precision:** In a few places, the narrative could be tightened to clearly distinguish between perceived improvements and objectively measured service outcomes. Minor wording adjustments would address this without changing the substance of the findings.<br>• **Minor editorial refinements-** Address small grammatical issues and ensure consistent	This is addressed on lines from 452 to 462.<br><br>In line with the qualitative nature of the study, the Results section was revised to emphasize digital health design and implementation lessons, focusing on perceived improvements rather than objectively measured outcomes.<br><br>We used average to be consistent.

terminology (e.g., use of “mean” vs. “average”) would improve readability.	
**Reviewer 3**	
• The manuscript is well written with great strengths such as the qualitative method used and the multicenter study conducted as well. However, since the journal is related to digital health, it would have been prudent to disclose the hardware specifications and the software architecture.	This is addressed from lines from 99 to 102.
• Authors should provide a brief section on transition strategies for the increased workload and the inclusion of stakeholders, such as the health Ministry, since the cost implications would have to be considered in the national budget.	These are addressed on lines from 452 to 462.
• There was also no explanation on why some participants did not receive text messages, any reasons for that, or whether this would affect a nationwide rollout.	Following a consultation with the technical team, a configuration issue in the digital system was preventing SMS reminders from being sent to pregnant women. However, following a data migration process, all women began receiving SMS reminders.
